# Sex difference in the associations among risk factors with gastroesophageal reflux disease in a large Taiwanese population study

**DOI:** 10.1186/s12876-024-03254-3

**Published:** 2024-05-15

**Authors:** Chien-Chieh Lin, Jiun-Hung Geng, Pei-Yu Wu, Jiun-Chi Huang, Huang-Ming Hu, Szu-Chia Chen, Chao-Hung Kuo

**Affiliations:** 1 Department of Internal Medicine, Kaohsiung Medical University Hospital, Kaohsiung Medical University, Kaohsiung, 807 Taiwan; 2 Department of Internal Medicine, Division of Gastroenterology, Kaohsiung Medical University Hospital, Kaohsiung Medical University, Kaohsiung, 807 Taiwan; 3 Department of Urology, Kaohsiung Municipal Siaogang Hospital, Kaohsiung Medical University Hospital, Kaohsiung Medical University, Kaohsiung, 812 Taiwan; 4 Department of Urology, Kaohsiung Medical University Hospital, Kaohsiung Medical University, Kaohsiung, 807 Taiwan; 5 Department of Internal Medicine, Kaohsiung Municipal Siaogang Hospital, Kaohsiung Medical University, 482, Shan-Ming Rd., Siaogang Dist., Kaohsiung, 812 Taiwan R.O.C.; 6 Department of Internal Medicine, Division of Nephrology, Kaohsiung Medical University Hospital, Kaohsiung Medical University, Kaohsiung, 807 Taiwan, Republic of China; 7 Faculty of Medicine, College of Medicine, Kaohsiung Medical University, Kaohsiung, 807 Taiwan

**Keywords:** Sex difference, Gastroesophageal reflux disease, Taiwan Biobank

## Abstract

**Background:**

Gastroesophageal reflux disease (GERD) is a common global health issue. Previous studies have revealed a higher prevalence of GERD in females than in males, however few studies have investigated sex differences in the risk factors associated with GERD. Therefore, the aim of this population-based study was to examine sex differences in the risk factors for GERD in a large cohort of over 120,000 Taiwanese participants.

**Methods:**

We enrolled 121,583 participants (male: 43,698; female: 77,885; mean age 49.9 ± 11.0 years) from the Taiwan Biobank. The presence of GERD was ascertained using self-reported questionnaires. Sex differences in the risk factors associated with GERD were examined using multivariable logistic regression analysis.

**Results:**

The overall prevalence of GERD was 13.7%, including 13.0% in the male participants and 14.1% in the female participants (*p* < 0.001). Multivariable analysis showed that older age, hypertension, smoking history, alcohol history, low fasting glucose, and low uric acid were significantly associated with GERD in the male participants. In the female participants, older age, diabetes, hypertension, smoking history, alcohol history, low systolic blood pressure, low fasting glucose, high hemoglobin, high total cholesterol, low high-density lipoprotein cholesterol (HDL-C), low low-density lipoprotein cholesterol, and low uric acid were significantly associated with GERD. Significant interactions were found between sex and age (*p* < 0.001), diabetes (*p* < 0.001), smoking history (*p* < 0.001), fasting glucose (*p* = 0.002), triglycerides (*p* = 0.001), HDL-C (*p* = 0.001), and estimated glomerular filtration rate (*p* = 0.002) on GERD.

**Conclusions:**

Our results showed a higher prevalence of GERD among females compared to males. Furthermore, sex differences were identified in the risk factors associated with GERD, and older age, diabetes, smoking history, and low HDL-C were more closely related to GERD in females than in males.

## Background

Gastroesophageal reflux disease (GERD) is a common global health issue, with a prevalence of approximately 10% to 20% in Western countries and about 2.5% to 17% in Asia [1–5]. In Taiwan, the prevalence of GERD has been estimated to be around 25% in the community [6]. Female sex and an age from 40–59 years are both known risk factors related to the development of GERD [6]. The pathogenesis of GERD is considered to be multifactorial, including transient lower esophageal sphincter relaxation and other lower esophageal sphincter pressure malfunctions, leading to reflux of acid, bile, pepsin and pancreatic enzymes into the esophagus causing mucosal damage [7]. Other etiologies of GERD such as hiatal hernia, impaired esophageal clearance, delayed gastric emptying and impaired mucosal defensive factors have also been reported [8–10]. Symptoms of GERD such as heartburn, regurgitation, belching, fullness sensation affect females more than males, while pathologic changes are more common in males than in females [11, 12]. Esophageal sensitivity can also differ between males and females, and this may explain the difference in symptoms [13]. On the other hand, the anti-inflammatory and mucosal protective effects of estrogen may play a role against acid reflux-induced mucosal damage [14, 15]. Complication from GERD such as Barrett's esophagus, erosive esophagitis, and chronic laryngitis may induce further structural stricture and increase the risk of esophageal adenocarcinoma [11]. Hence, identifying risk factors which may be associated with GERD and the sex differences among these risk factors is important to decrease the burden on healthcare systems and develop new treatments to improve patient care.

Sex differences have been observed in many diseases, including cardiovascular diseases, cancers and liver diseases, and these differences have an important influence on the clinical presentation, progression, and response to management [16]. The mechanisms behind sex differences and sexual dimorphism are considered to be linked to sex hormones [17]. Hung et al. evaluated the prevalence and risk factors for GERD in 1238 people in the general population in Taiwan, and found that female sex was an independent risk factor related to the development of GERD, with an odds ratio (OR) of 1.71 [6]. Nevertheless, few studies have investigated sex differences in the risk factors associated with GERD [18–20]. In S Nasseri-Moghaddam’s study, frequent GERD was seen in 18.2%. Female sex (OR: 1.55) was a risk factor for GERD. However, the study didn’t separately compare risk factors of different sex with the association of GERD [20]. In J C Chiocca’s study, no statistically significant differences between sexes were observed. Thus, no further comparing risk factors of different genders with the association of GERD [18]. Shyam Menon et al. evaluated 154 406 upper gastrointestinal endoscopies, 24 240 (15.7%) patients had reflux esophagitis. The incidence of reflux esophagitis increased was more marked in women with increasing age, compared with men. However, this study only investigated the GERD relationship with age and sex. Other risk factors were not surveyed in this article [19]. Therefore, the aim of this population-based study was to examine sex differences in the risk factors for GERD in a large cohort of over 120,000 Taiwanese participants.

## Methods

### The Taiwan Biobank (TWB)

Due to the increasingly aged population in Taiwan, the TWB was established by the Ministry of Health and Welfare for the promotion of health services and prevention of chronic diseases. The TWB enrolls cancer-free members of the general community between the ages of 30 and 70 years, and records lifestyle factor, genomic and medical data [21–23]. The detailed information could be obtained from the official website: https://www.twbiobank.org.tw/. The Ethics and Governance Council of the TWB and Institutional Review Board on Biomedical Science Research, Academia Sinica, Taiwan granted ethical approval.

On enrollment, basic and medical data are collected from all participants, including age, presence of diabetes mellitus (DM) and hypertension, weight and height. Laboratory analysis of serum is also performed at baseline and after an 8-h fast (COBAS Integra 400, Roche Diagnostics GmbH, D-68298 Mannheim, Germany), and data on fasting glucose, hemoglobin, triglycerides, total cholesterol, high- and low-density lipoprotein cholesterol (HDL-C/LDL-C), estimated glomerular filtration rate (eGFR) (calculated as reported previously [24]), uric acid and creatinine (calculated as reported previously [24]) are recorded.

Systolic and diastolic blood pressure (BPs) are also measured using an automated BP device by a nurse, and the average of three measurements was used in our analysis. We also recorded details of exercise habits, and a minimum of three 30-min sessions in 1 week was defined as “regular exercise” according to current guidelines in Taiwan [25]. This study was conducted in accordance with the Helsinki Declaration, and approved by the Institutional Review Board of Kaohsiung Medical University Hospital (KMUHIRB-E(I)-20,210,058).

### Sample population and sample size

Participants between 30–70 years of age without a personal history of cancer, recruited between 2012 and 2018 in the TWB. All participants were enrolled. A total of 121,583 participants (male: 43,698; female: 77,885; mean age 49.9 ± 11.0 years) were included to analyze sex differences among the risk factors for GERD. Those who reported a history of GERD after completing a questionnaire were classified into the GERD group, and those who did were classified into the non-GERD group.

### Statistical analysis

Continuous data are shown in mean ± (SD), with categorical variables in number (%). Comparisons of continuous data were made using the independent t test, and comparisons of categorical data were made using the chi-square test. Associations between GERD and the studied risk factors in the male and female participants were examined using multivariable logistic regression analysis. An interaction *p* and β in logistic analysis: Model disease (y) =  × 1 +  × 2 +  × 1 ×  × 2 + covariates. × 1 ×  × 2 was the interaction term, where y = GERD and its components; × 1 = sex; × 2 = each variable; covariates = age, DM and hypertension, smoking, alcohol and regular exercise habits, systolic and diastolic BPs, body mass index (BMI), fasting glucose, hemoglobin, triglycerides, total cholesterol, HDL-C, LDL-C, eGFR and uric acid. From the value of β, we can know which sex is more closely associated with GERD. If β > 0, this factor is more closely associated with male (OR > 1), and female (OR < 1); if β < 0, this factor is more closely associated with female (OR > 1) and male (OR < 1). In our database, we set male = 1, female = 0. We used SPSS for the analysis (v19, IBM Inc., Armonk, NY), and two-tailed *p* values < 0.05 were considered to be significant.

## Results

Overall, 13.7% of the 121,583 enrolled participants had GERD, including 13.0% of the male participants and 14.1% of the female participants (*p* < 0.001).

### Comparison of the participants with and without GERD

Table 1 shows comparisons of the clinical characteristics between the GERD and non-GERD groups. The GERD group was older, had a higher proportion of female participants, and higher prevalence of DM, hypertension, smoking, alcohol and regular exercise habits, higher fasting glucose, triglycerides, total cholesterol and LDL-C, and lower eGFR and uric acid than the non-GERD group.

**Table 1:** Clinical characteristics of the study participants classified by the presence of GERD

Characteristics		GERD (*n* = 121,583)	
	GERD (-) (*n* = 104,919)	GERD ( +) (*n* = 16,664)	*p*
Age (year)	49.6 ± 11.0	51.9 ± 10.4	< 0.001
Male sex (%)	36.2	34.2	< 0.001
DM (%)	5.0	6.3	< 0.001
Hypertension (%)	11.7	15.3	< 0.001
Smoking history (%)	26.9	29.3	< 0.001
Alcohol history (%)	8.3	9.8	< 0.001
Regular exercise habits (%)	40.2	43.0	< 0.001
Systolic BP (mmHg)	120.4 ± 18.8	120.7 ± 17.8	0.102
Diastolic BP (mmHg)	73.8 ± 11.5	73.8 ± 10.9	0.562
BMI (kg/m^2^)	24.2 ± 3.8	24.2 ± 3.8	0.277
Laboratory parameters			
Fasting glucose (mg/dL)	95.9 ± 20.9	96.4 ± 19.7	0.002
Hemoglobin (g/dL)	13.8 ± 1.6	13.8 ± 1.5	0.083
Triglyceride (mg/dL)	115.1 ± 94.3	119.0 ± 92.3	< 0.001
Total cholesterol (mg/dL)	195.4 ± 35.9	197.4 ± 35.2	< 0.001
HDL-C (mg/dL)	54.6 ± 13.4	54.6 ± 13.6	0.668
LDL-C (mg/dL)	120.8 ± 31.8	121.8 ± 31.3	< 0.001
eGFR (mL/min/1.73 m^2^)	103.5 ± 23.9	102.1 ± 23.7	< 0.001
Uric acid (mg/dL)	5.43 ± 1.43	5.39 ± 1.39	0.002

*Abbreviations*: *GERD* Gastroesophageal reflux disease, *DM* Diabetes mellitus, *BP* Blood pressure, *BMI* Body mass index, *HDL-C* High-density lipoprotein cholesterol, *LDL-C* Low-density lipoprotein cholesterol, *eGFR* Estimated glomerular filtration rate

### Determinants of GERD

Multivariable analysis with adjustments for the covariates listed in the *Statistical analysis* section showed significant associations between GERD with female sex (male *vs.* female; OR = 0.786; 95% confidence interval = 0.744–0.829), older age, hypertension, smoking history, alcohol history, low systolic BP, low fasting glucose, high hemoglobin, low uric acid (all *p* < 0.001), DM (*p* = 0.001), high BMI (*p* = 0.040), high total cholesterol (*p* = 0.001), low HDL-C (*p* = 0.003), and low LDL-C (*p* = 0.009) (Table 2).

**Table 2:** Determinants for GERD using multivariable logistic regression analysis

Variables	Multivariable (GERD)	
	Odds ratio (95% CI)	*p*
Age (per 1 year)	1.021 (1.020–1.023)	< 0.001
Male (*vs.* female)	0.786 (0.744–0.829)	< 0.001
DM	1.155 (1.066–1.251)	< 0.001
Hypertension	1.241 (1.178–1.307)	< 0.001
Smoking history	1.237 (1.184–1.293)	< 0.001
Alcohol history	1.177 (1.108–1.251)	< 0.001
Regular exercise habits	0.984 (0.950–1.020)	0.375
Systolic BP (per 1 mmHg)	0.994 (0.992–0.995)	< 0.001
Diastolic BP (per 1 mmHg)	1.002 (1.000–1.005)	0.070
BMI (per 1 kg/m^2^)	1.006 (1.000–1.011)	0.040
Laboratory parameters		
Fasting glucose (per 1 mg/dL)	0.998 (0.997–0.999)	< 0.001
Hemoglobin (per 1 g/dL)	1.035 (1.020–1.049)	< 0.001
Triglyceride (per 10 mg/dL)	0.999 (0.996–1.002)	0.627
Total cholesterol (per 10 mg/dL)	1.032 (1.013–1.051)	0.001
HDL-C (per 1 mg/dL)	0.996 (0.994–0.999)	0.003
LDL-C (per 1 mg/dL)	0.997 (0.996–0.999)	0.009
eGFR (per 1 mL/min/1.73 m^2^)	0.999 (0.999–1.000)	0.115
Uric acid (per 1 mg/dL)	0.962 (0.947–0.977)	< 0.001

Values expressed as odds ratio and 95% confidence interval (CI). Abbreviations are the same as in Table 1

Adjusted for age, sex, DM and hypertension, smoking and alcohol history, regular exercise habit, systolic and diastolic BPs, BMI, fasting glucose, hemoglobin, triglyceride, total cholesterol, HDL-C, LDL-C, eGFR and uric acid

### Comparison of the male and female participants

Compared to the male participants, the female participants had lower prevalence of DM and hypertension, higher prevalence of GERD, and lower prevalence of smoking, alcohol and regular exercise habits, higher total cholesterol, HDL-C and eGFR, and lower systolic and diastolic BPs, BMI, fasting glucose, hemoglobin, triglycerides, LDL-C, and uric acid (Table 3).

**Table 3:** Clinical characteristics of the study participants classified by sex

Characteristics	Male (*n* = 43,698)	Female (*n* = 77,885)	*p*
Age (year)	49.9 ± 11.4	49.9 ± 10.7	0.826
DM (%)	6.8	4.2	< 0.001
Hypertension (%)	16.8	9.7	< 0.001
GERD (%)	13	14.1	< 0.001
Smoking history (%)	57.4	10.4	< 0.001
Alcohol history (%)	18.7	2.8	< 0.001
Regular exercise habits (%)	42.4	39.5	< 0.001
Systolic BP (mmHg)	126.3 ± 17.3	117.1 ± 18.6	< 0.001
Diastolic BP (mmHg)	78.5 ± 11.1	71.2 ± 10.7	< 0.001
BMI (kg/m^2^)	25.4 ± 3.6	23.6 ± 3.8	< 0.001
Laboratory parameters			
Fasting glucose (mg/dL)	99.3 ± 23.4	94.0 ± 18.8	< 0.001
Hemoglobin (g/dL)	15.1 ± 1.2	13.0 ± 1.3	< 0.001
Triglyceride (mg/dL)	137.9 ± 117.9	103.1 ± 74.6	< 0.001
Total cholesterol (mg/dL)	191.9 ± 35.1	197.8 ± 36.1	< 0.001
HDL-C (mg/dL)	48.0 ± 11.1	58.2 ± 13.3	< 0.001
LDL-C (mg/dL)	121.7 ± 31.5	120.5 ± 31.9	< 0.001
eGFR (mL/min/1.73 m^2^)	93.8 ± 19.5	108.6 ± 24.5	< 0.001
Uric acid (mg/dL)	6.4 ± 1.4	4.9 ± 1.1	< 0.001

Abbreviations are the same as in Table 1

### Comparisons of the male and female participants with and without GERD

The male participants with GERD were older, had higher prevalence of hypertension, smoking, alcohol and regular exercise habits, lower prevalence of hyperuricemia, and lower diastolic BP, BMI, uric acid and eGFR than the male participants without GERD (Table 4). The female participants with GERD were older, had higher prevalence of DM, hypertension, alcohol, smoking and regular exercise habits, higher systolic BP, BMI, fasting glucose, hemoglobin, triglycerides, total cholesterol, LDL-C and uric acid, and lower HDL-C and eGFR than the female participants without GERD (Table 4).

**Table 4:** Clinical characteristics of the study participants classified by the presence of different sex and GERD

Characteristics	Male (*n* = 43,698)			Female (*n* = 77,885)		
	GERD (-) (*n* = 38,004)	GERD ( +) (*n* = 5694)	*p*	GERD (-) (*n* = 66,915)	GERD ( +) (*n* = 10,970)	*p*
Age (year)	49.6 ± 11.4	51.7 ± 10.7	< 0.001	49.5 ± 10.8	52.0 ± 10.2	< 0.001
DM (%)	6.7	7.3	0.086	4.0	5.8	< 0.001
Hypertension (%)	16.2	20.8	< 0.001	9.2	12.5	< 0.001
Smoke history (%)	56.7	62.1	< 0.001	10.0	12.3	< 0.001
Alcohol history (%)	18.2	22.3	< 0.001	2.7	3.3	< 0.001
Regular exercise habits (%)	42.0	44.5	0.001	39.1	42.3	< 0.001
Systolic BP (mmHg)	126.3 ± 17.5	126.3 ± 16.4	0.949	117.0 ± 18.7	117.7 ± 17.8	< 0.001
Diastolic BP (mmHg)	78.4 ± 11.2	78.5 ± 10.4	0.563	71.2 ± 10.8	71.3 ± 10.3	0.345
BMI (kg/m^2^)	25.4 ± 3.6	25.4 ± 3.6	0.929	23.6 ± 3.7	23.7 ± 3.7	0.004
Laboratory parameters						
Fasting glucose (mg/dL)	99.3 ± 23.6	99.4 ± 21.9	0.889	93.9 ± 10.9	94.8 ± 18.3	< 0.001
Hemoglobin (g/dL)	15.1 ± 1.2	15.1 ± 1.2	0.954	13.0 ± 1.3	13.1 ± 1.2	< 0.001
Triglyceride (mg/dL)	137.7 ± 119.4	138.8 ± 106.9	0.524	102.2 ± 73.3	108.7 ± 81.8	< 0.001
Total cholesterol (mg/dL)	191.8 ± 35.2	192.6 ± 34.7	0.109	197.4 ± 36.2	199.9 ± 35.2	< 0.001
HDL-C (mg/dL)	48.0 ± 11.1	48.1 ± 11.3	0.305	58.3 ± 13.2	58.0 ± 13.4	0.016
LDL-C (mg/dL)	121.7 ± 31.5	121.8 ± 31.6	0.765	120.3 ± 32.0	121.7 ± 31.1	< 0.001
eGFR (mL/min/1.73 m^2^)	93.9 ± 19.4	93.1 ± 19.8	0.005	108.9 ± 24.5	106.7 ± 24.2	< 0.001
Uric acid (mg/dL)	6.44 ± 1.37	6.35 ± 1.35	< 0.001	4.86 ± 1.12	4.90 ± 1.13	< 0.001

Abbreviations are the same as in Table 1

### Associations and interactions among the risk factors for GERD in the male and female participants

Table 5 shows the association and interaction of factors with GERD in different sex using multivariable logistic regression analysis. Regarding the associations and interactions among the risk factors for GERD in the male and female participants, multivariable logistic regression analysis with adjustments for the covariates listed in the *Statistical analysis* section showed significant associations between GERD with older age, hypertension, smoking history, alcohol history, low uric acid (all *p* < 0.001) and low fasting glucose (*p* = 0.004) in the male participants (Table 5). In the female participants, older age, DM, hypertension, smoking history, low systolic BP (all *p* < 0.001), low uric acid (*p* = 0.001), alcohol history (*p* = 0.033), low fasting glucose (*p* = 0.002), high hemoglobin (*p* = 0.001), high total cholesterol (*p* = 0.015), low HDL-C (*p* = 0.008), and low LDL-C (*p* = 0.024) were significantly associated with GERD.

**Table 5:** Association and interaction of factors with GERD in different sex using multivariable logistic regression analysis

Characteristics	Male (*n* = 43,698)			Female (*n* = 77,885)				Interaction
	OR	95% CI	*p*	OR	95% CI	*p*	**β**	*p*
Age (per 1 year)	1.017	1.013–1.020	< 0.001	1.025	1.023–1.028	< 0.001	-0.009	< 0.001
DM	0.986	0.869–1.118	0.828	1.301	1.173–1.443	< 0.001	-0.365	< 0.001
Hypertension	1.272	1.177–1.376	< 0.001	1.238	1.154–1.328	< 0.001	-0.090	0.062
Smoking history	1.161	1.094–1.233	< 0.001	1.386	1.299–1.480	< 0.001	-0.170	< 0.001
Alcohol history	1.213	1.129–1.303	< 0.001	1.137	1.010–1.279	0.033	-0.001	0.986
Regular exercise habits	0.989	0.932–1.051	0.727	0.977	0.935–1.021	0.311	-0.037	0.301
Systolic BP (per 1 mmHg)	0.993	0.991–0.996	< 0.001	0.993	0.991–0.995	< 0.001	-0.001	0.150
Diastolic BP (per 1 mmHg)	1.004	1.000–1.008	0.051	1.002	0.999–1.005	0.207	0	0.960
BMI (per 1 kg/m^2^)	1.007	0.997–1.016	0.165	1.004	0.998–1.011	0.194	-0.005	0.291
Laboratory parameters								
Fasting glucose (per 1 mg/dL)	0.998	0.996–0.999	0.004	0.998	0.997–0.999	0.002	-0.003	0.002
Hemoglobin (per 1 g/dL)	1.023	0.998–1.049	0.073	1.031	1.013–1.049	0.001	-0.005	0.744
Triglyceride (per 10 mg/dL)	0.998	0.993–1.003	0.481	1.001	0.997–1.006	0.571	-0.006	0.001
Total cholesterol (per 10 mg/dL)	1.027	0.997–1.059	0.079	1.031	1.006–1.056	0.015	0.004	0.474
HDL-C (per 1 mg/dL)	0.999	0.995–1.003	0.550	0.996	0.992–0.999	0.008	0.005	0.001
LDL-C (per 1 mg/dL)	0.998	0.995–1.001	0.241	0.997	0.995–1.000	0.024	0.001	0.178
eGFR (per 1 mL/min/1.73 m^2^)	1.000	0.999–1.002	0.820	0.999	0.998–1.000	0.053	0.003	0.002
Uric acid (per 1 mg/dL)	0.949	0.927–0.970	< 0.001	0.963	0.943–0.984	0.001	-0.027	0.057

Values expressed as odds ratio (OR) and 95% confidence interval (CI). Abbreviations are the same as in Table 1

Adjusted for age, DM and hypertension, smoking and alcohol history, regular exercise habit, systolic and diastolic BPs, BMI, fasting glucose, hemoglobin, triglyceride, total cholesterol, HDL-C, LDL-C, eGFR and uric acid

Significant interactions were found between sex and the following factors on GERD: age (*p* < 0.001), DM (*p* < 0.001), smoking history (*p* < 0.001), fasting glucose (*p* = 0.002), triglycerides (*p* = 0.001), HDL-C (*p* = 0.001), and eGFR (*p* = 0.002).

## Discussion

In this study, we analyzed sex differences in the risk factors associated with GERD in a large Taiwanese population. We found that the female participants had a higher prevalence of GERD than the male participants, and that the risk factors associated with GERD in the female participants were older age, DM, hypertension, smoking and alcohol history, low systolic BP, high hemoglobin, low fasting glucose, high total cholesterol, low HDL-C, low LDL-C and low uric acid. On the other hand, the risk factors associated with GERD in the male participants were older age, hypertension, smoking and alcohol history, low systolic BP, low fasting glucose and low uric acid. Furthermore, there were sex differences in the risk factors associated with GERD, including age, DM, smoking history, fasting glucose, triglyceride, HDL-C and eGFR. Among them, older age, DM, smoking history, and low HDL-C were more closely related to GERD in the females than in the males.

Our finding of a higher prevalence of GERD in the female participants than in the male participants is consistent with the study by Nasseri-Moghaddam et al., who also reported that female participants had a higher prevalence of GERD (OR = 1.55 in frequent GERD, OR = 1.24 in infrequent GERD) in a questionnaire survey conducted in Iran [20]. According to their study, female sex was independently associated with frequent GERD. A possible explanation for this finding is that risk factors associated with GERD such as a higher BMI, less education, smoking, and use of non-steroidal anti-inflammatory drugs were also more prevalent in females, and these factors may have contributed to the higher prevalence of GERD in this population [20]. Another population-based study conducted in urban Brazil by Moraes-Filho et al. also demonstrated that GERD symptoms including heartburn and regurgitation were more frequently found in females than in males [26]. In addition, they found that the occurrence of GERD increased with emotional distress which is significant linked with GERD and women. In Taiwan, women were significantly associated with depression compared to men [27]. Our study did not survey the relationship between emotional condition with GERD and sex. However, the significant higher prevalence of depression in women than in men might contribute to the higher prevalence of GERD in females. The anti-inflammatory effect of estrogen had been shown in animal models, and this effect has been shown to enhance esophageal mucosal resistance to gastric acid injury and nitric oxide-induced tissue damage [28, 29]. In a study of male rats, exogenous nitric oxide treatment was shown to lead to the development of severe esophageal ulcers and inflammation, characterized by the infiltration of lymphocytes. In contrast, only mild tissue damage was observed in female rats under similar conditions [28]. These mechanisms may at least partially explain the sex differences in GERD.

We also found that older age was more closely related to GERD in females than in males. Menon et al. reported that the incidence of GERD increased with age, but that this phenomenon was more obvious in females than in males [19]. Braniste et al. postulated that the decrease in serum estrogen levels resulting from aging could compromise epithelial integrity and induce bacterial translocation, particularly post menopause in females [30, 31]. This may then result in an increased incidence of GERD and also more severe reflux symptoms [15, 32]. Another factor associated with reflux symptoms, especially in nonerosive reflux disease, is visceral hypersensitivity. This may be caused by the upregulation of certain molecules in the esophageal mucosal, such as transient receptor potential cation channel subfamily V member 1 [33, 34]. Females have been shown to have higher sensitivity to mechanical stimuli of pain and larger referred pain areas in the esophagus than males [35]. Therefore, we hypothesize that the age-associated decrease in serum estrogen levels may explain why older age was more closely related to GERD in females.

Another interesting finding of this study is that DM was more closely related to GERD in females than in males. The association between DM and upper gastroenterology symptoms such as GERD has been reported in many studies [36, 37]. Lower esophageal contraction strength, frequency of peristaltic waves, and pressure in the lower esophageal sphincter along with atypical gastroesophageal reflux may contribute to this phenomenon [38, 39]. Decreased density and anomalous morphology of nerve fibers in the gastric mucosa of individuals with DM also play a role in peristalsis dysfunction [40, 41]. A meta-analysis of trends in the prevalence and incidence of DM in Taiwan from 2000–2014 disclosed that females with DM had a higher percentage of > 10 years lived with disability than males [42, 43]. We hypothesize that the longer patients have DM, the worse esophageal nerve damage will be. This pathophysiology may then worsen GERD in female patients with DM. In addition, female patients with DM are also associated with a higher incidence of other comorbidities. For example, female DM patients are more likely to develop metabolic syndrome (MetS) compared to male DM patients [44–46]. A possible explanation for this phenomenon is that after menopause, the decline in estrogen levels leads to changes in body composition and metabolism, including an increase in body fat and a decrease in lean muscle mass [47]. This evolutionary energy conservation regulation may increase the risk of MetS in modern female DM patients, and consequently increase the prevalence of GERD.

Another key finding of this study is that smoking was more closely related to GERD in females than in males. Smoking can induce GERD by nicotine blocking cholinergic receptors, which in turn reduces pressure in the lower esophagus [48, 49]. In addition, smoking causes a reduction in the rate of salivary secretion and decrease in salivary bicarbonate concentration, which then reduces acid clearance time [50]. Nilsson et al. conducted a large population-based study (43,363 cases) in Norway, and found that tobacco smoking was highly associated with GERD, but with no sex difference [51]. In our study, however, we found that the female smokers had a greater OR than the male smokers for GERD. Smoking has also been associated with sex differences in other health issues. For instance, women who smoke have been associated with a higher risk of lung cancer than men with similar smoking exposure [52]. Sex differences in the expressions of some somatic gene mutations in Kirsten rat sarcoma viral oncogene or epidermal growth factor receptor have been shown to induce higher levels of DNA adducts in women than in men [53]. Another example of a sex difference in smoking-related comorbidity is coronary artery disease, and Huxley et al. reported that compared with nonsmokers, female smokers had a 25% greater relative risk of coronary artery disease than male smokers [54]. The protective effect of estrogen on plaque rupture or erosions is considered to be more prominent in women than in men [55]. In addition, estrogen may interfere with the accumulation of foam cells in coronary plaques [55]. Nevertheless, further studies are needed to clarify the mechanisms by which smoking causes a higher prevalence of GERD in women than in men.

Our results also showed that a low HDL-C level was more closely related to GERD in females than in males. In a study by Hsieh et al. of 4895 patients who received upper gastrointestinal endoscopy in Taiwan, a low HDL-C level was not significantly associated with GERD [56]. In addition, a Korean population study analyzed a total of 6082 subjects with MetS, and also found no significant association between HDL-C and GERD [57]. A decreased level of serum HDL-C has been associated with long-term Helicobacter pylori (H. pylori) infection, which may also be associated with cardiovascular disease [58]. It has been shown that chronic H. pylori infection is not associated with GERD or even inversely correlated with the severity of reflux esophagitis [59, 60]. However, symptoms caused by chronic H. pylori infection such as epigastric pain, nausea, vomiting, and delayed gastric emptying may mimic the symptoms of GERD. Chen et al. recently reported an updated analysis on the prevalence of H. pylori infection in Taiwan, and reported that females had a significantly higher OR of H. pylori infection [61]. Because our data were acquired by self-reported questionnaire, it is possible that some of the GERD patients had concomitant H. pylori infection. HDL-C is currently considered to be not only associated with cholesterol transportation, but also with anti-inflammation, cellular antioxidant activity and immunomodulation [62, 63]. Notably, serum estrogen has been demonstrated to be inversely proportional to serum HDL-C level, and especially in menopausal women [64]. We postulate that the decrease in estrogen level in menopause may induce chronic inflammation, which consequently damages the gastric mucosa, lower esophageal sphincter and enteric nervous system, and exacerbates GERD.

The strength of this research is the inclusion of large cohort of community-dwelling participants and the comprehensive analysis of sex differences in the risk factors associated with GERD. Nevertheless, several limitations should also be noted. First, we did not evaluate the duration of GERD in this cross-sectional study, and hence we could not evaluate causal relationships. Further longitudinal studies are warranted to investigate the risk of incident GERD. Second, we did not validate the self-reported presence of GERD, and we could not analyze the type and severity of GERD. In addition, no further symptoms and/or signs were evaluated. Therefore, we could analyze the associated factors with symptoms/signs of GERD. Further research is needed to explore the sex difference in the associations among risk factors with the type and severity of GERD. Nevertheless, a previous study demonstrated fair agreement between claims data and self-reported diseases in Taiwan. Finally, the Chinese ethnicity of our participants may limit the findings to other groups.

## Conclusion

In conclusion, our results showed a higher prevalence of GERD in females than in males in this large Taiwanese cohort. Furthermore, there were sex differences in the risk factors associated with GERD, and older age, DM, smoking history, and low HDL-C were more closely related to GERD in females than in males.

## Data Availability

The data underlying this study are from the Taiwan Biobank Database. Due to restrictions placed on the data by the Personal Information Protection Act of Taiwan, the minimal data set cannot be made publicly available. Data may be available upon request to interested researchers. Please send data requests to: Szu-Chia Chen, PhD, MD. Division of Nephrology, Department of Internal Medicine, Kaohsiung Medical University Hospital, Kaohsiung Medical University.

## Abbreviations

BMI: Body mass index
BP: Blood pressure
DM: Diabetes mellitus
eGFR: Estimated glomerular filtration rate
GERD: Gastroesophageal reflux disease
HDL-C: High-density lipoprotein cholesterol
H. pylori: Helicobacter pylori
LDL-C: Low-density lipoprotein cholesterol
OR: Odds ratio
TWB: Taiwan Biobank
